# The effect of reduced RDI of chemotherapy on the outcome of breast cancer patients

**DOI:** 10.1038/s41598-020-70187-8

**Published:** 2020-08-06

**Authors:** Wanwan Qi, Xiaoyi Wang, Lu Gan, Yunhai Li, Hongyuan Li, Qiao Cheng

**Affiliations:** 1 Department of Breast and Endocrine Surgery, The First Affiliated Hospital of Chongqing Medical University, No. 1 Youyi Road, Yuzhong District, Chongqing, 400016 P. R. China; 2 Department of Oncology, The First Affiliated Hospital of Chongqing Medical University, No. 1 Youyi Road, Yuzhong District, Chongqing, 400016 P. R. China

**Keywords:** Cancer, Diseases, Medical research

## Abstract

The aims of this study were to investigate the impact of the relative dose intensity (RDI) of chemotherapy on disease-free survival (DFS) and overall survival (OS), to identify the optimal RDI cut-off points with the docetaxel, epirubicin and cyclophosphamide (TEC) regimen for stage I–III breast cancer patients and to explore the adverse events in these patients. To achieve this, we performed a retrospective analysis of breast cancer patients treated at the First Affiliated Hospital of Chongqing Medical University in 2011. The results showed that among 293 patients with the TEC regimen, 85% and 80% were the cut-off points at which a high RDI was associated with better overall survival (HR = 2.04; 95% CI 1.13, 3.70; p = 0.02) and disease-free survival (HR = 1.97; 95% CI 1.14–3.42; p = 0.02), respectively. Among 169 HR(+) patients, 80% was the cut-off point for DFS (HR = 2.33; 95% CI 1.07–5.08; p = 0.03), and 85% was the cut-off point for OS (HR = 3.00; 95% CI 1.24–7.26; p = 0.02). Among 105 HR(−) patients, 80% was the cut-off point for OS (HR = 2.86; 95% CI 1.05–7.80; p = 0.04). Of 293 patients, neutropenia, nausea, and vomiting were found to be correlated with the level of RDI. In conclusion, a higher RDI of chemotherapy is associated with better survival but with a higher probability of causing adverse events. To optimize survival benefits, the RDI should be maintained ≥ 85% for HR(+) patients and ≥ 80% for HR(−) patients.

## Introduction

The current standard of care for breast cancer includes breast surgery, local radiation therapy, systemic adjuvant and neoadjuvant chemotherapy, endocrine therapy and target therapy. Studies have shown the effect of chemotherapy on improving breast cancer survival^1–3^. An increased benefit with anthracycline-containing regimens, which have been used increasingly over the past decade, has been recommended by The Early Breast Cancer Trialists’ Collaborative Group^4^. In addition, support for the adjuvant use of taxanes after an anthracycline-containing regimen was provided by Henderson et al.’s report (Intergroup Study 0148)^1,5^. As an increasing number of trials have suggested an increased benefit with anthracycline and taxane-containing regimens, doctors have used taxanes combined with anthracycline as a classical regimen. The most famous clinical trial BCIRG005 suggested that patients receiving both doxorubicin and cyclophosphamide followed by docetaxel (AC-T) and docetaxel in combination with doxorubicin and cyclophosphamide (TAC) had a 79.0% 5-year disease-free survival rate. The 5-year overall survival rates were 89.0% and 88.0%, respectively^6^. The 10-year overall survival rate was 79.9% in the AC-T arm and 78.9% in the TAC arm. The AC-T arm had a 66.5% 10-year disease-free survival rate, and the rate of the TAC arm was 66.3%^7^. The survival benefits of the two regimens were similar, but the incidence of grade 3/4 adverse events in the blood system was lower in the AC-T regimen than in the TAC regimen^6^. However, the TAC regimen with G-CSF support provided a shorter duration of adjuvant therapy with less toxicity and was proven by the clinical trial BCIRG005^7^. The TAC regimen was the most popular regimen in China years ago and is still widely used, especially in Western China.

Relative dose intensity (RDI) is an indicator used most frequently to measure and monitor the quality of chemotherapy. The relationship between chemotherapy dose intensity and clinical outcome has been studied in some trials. Most of the trials support the importance of sustaining full dose intensity in neoadjuvant and adjuvant chemotherapy, while others found the same clinical benefit of reducing RDI from the standard RDI for some regimens, and this remains the subject of much controversy. As a result of a 20-year follow-up, a randomized controlled trial suggested that early stage breast cancer (ESBC) patients with CMF regimens given an RDI ≥ 85% had a significantly better survival than those given regimens involving an RDI < 85%^8^. The survival benefits of receiving a higher RDI have been confirmed subsequently by several observational studies^9–11^. Therefore, to optimize survival benefits, maintaining the standard RDI has been supported by physicians and patients.

However, these trials were based on first-generation regimens such as cyclophosphamide, methotrexate and 5-fluorouracil (CMF) and patients from Western countries, and the optimal cut-off point of the RDI for the new generation of chemotherapy regimens in clinical practice and for Chinese patients is unknown. In China, especially Western China, where the living environment lags behind developed areas, medical and health conditions as well as lifestyles are different from those of developed areas and between Chinese and Western individuals. These various factors affect patients’ tolerance to chemotherapy. Many doctors tend to reduce the dose of chemotherapy empirically. It highlights the differences between theory and the real world. It is not certain whether high-dose chemotherapy will achieve the maximum benefit. It is unknown if 85% remains the optimal cut-off point of RDI for anthracyclines combined with taxane regimens in the current clinical practice.

Optimizing chemotherapy dose intensity is one strategy to reduce pain without decreasing survival outcomes. To date, little information is available on compliance with RDI recommendations for current chemotherapy regimens in clinical practice in Western China. Therefore, our study aimed to (1) investigate the impact of chemotherapy (combined neoadjuvant and adjuvant chemotherapies) RDI on disease-free and overall survival with a regimen containing anthracycline and taxane; (2) identify the optimal RDI cut-off points; and (3) investigate the adverse events of maintaining a high RDI compared with a low RDI for stage I–III breast cancer.

## Methods

### Data source and patient population

This study was approved by the Institutional Review Board of the First Affiliated Hospital of Chongqing Medical University and in accordance with the Declaration of Helsinki. Written informed consent was obtained from all individual participants or their family members. Data were collected by the electronic medical records system at our institute and telephone follow-up. Baseline patient demographics, disease-related variables (stage, ER/PR status), therapies and outcome analyses were carried out. Haematological and non-haematological toxicities were determined. Toxicity was graded in accordance with the Common Terminology Criteria for Adverse Events, version 4.0^12^. Patients were followed up until death or to early December 2018 if alive.

Eligible patients were female, diagnosed with American Joint Committee on Cancer (AJCC) (7th edition) stage I–III at the First Affiliated Hospital of Chongqing Medical University in 2011 and received chemotherapy with a TEC regimen (RDI could only be calculated for the standard regimens). Then, we divided the patients into two groups: HR+ (ER+ and/or PR+) and HR− (ER− and PR−).

Two survival outcomes were defined in this study: disease-free and overall survival. Disease-free survival (DFS) was defined as the time interval from surgery to tumor recurrence or metastasis. Overall survival (OS) was defined as the time interval from surgery to death or the last follow-up. The patients were followed up until death or the cutoff date (early December 2018).

### Chemotherapy relative dose intensity

The standard regimen for the adjuvant and neoadjuvant settings used in this study was recommended by the 2011 National Comprehensive Cancer Network (NCCN) guidelines for the treatment of breast cancer. Chemotherapy dose intensity is the amount of drug per unit (m^2^) of BSA per unit of time (week), calculated as the total dosage divided by the time interval (week) used to receive the dosage and patients’ BSA^13,14^. RDI is the ratio of dose intensity received by patients vs. dose intensity recommended by the standard regimens, ranging from 0 to 100%^15–18^. This method calculates d, the proportion of the planned dose received, t and the proportional time delay and combines the two to determine the RDI^19^. To identify the optimal cut-off point of the RDI, three cut-off points were predefined: 90, 85, and 80%. For each cut-off point, the disease-free and overall survival of patients receiving an RDI ≥ the evaluated cut-off point were compared with that of patients receiving an RDI < the evaluated cut-off point.

### Covariates

Covariates included age at diagnosis (≤ 49, 50–59, 60–69, > 69), menstrual status (pre-menopause, post-menopause), AJCC stage (I, II, III), tumour size (≤ 2.0, 2.0–5.0, > 5.0 cm), lymph node involvement (N0, N1, N2, N3), HR status (HR(+), HR(−)), surgical method (breast-conserving surgery, mastectomy), chemotherapy type (adjuvant, neoadjuvant), use of granulocyte-growth factors/cytokines (G-CSF) (yes, no), the use of hormone therapy (only for ER+/ PR+ patients; yes, no), with radiotherapy (yes, no), with comorbidity (yes, no), dose reduction (yes, no) and chemotherapy delays (yes, no). Since the status of HER2(2+) was unconfirmed because of the absence of fluorescence in situ hybridization (FISH), we divided the HER2/neu status of patients into positive, negative and unclear.

### Statistical analysis

The chi-square test was used to compare the distributions of categorical variables between comparison groups. A Cox proportional hazards model and Kaplan–Meier curve analysis were performed to compare the survival differences. The survival time was calculated from the date of first treatment to the date of death or the date of the last contact. Hazard ratios (HRs), 95% confidence intervals (CIs), and p values were reported for Cox proportional hazards models. The proportional hazard assumption was evaluated by adding an interaction term (product) of the time variable with the exposure variable (binary variable for reduced RDI) in the model^5,20^. A p-value smaller than 0.05 indicated the significance of the interaction term (i.e., the proportional hazard assumption was violated)^5,20^. All statistical analyses were performed with SPSS version 20.0 software. All methods in the current retrospective study were performed in accordance with relevant guidelines and regulations.

## Results

### Patient characteristics and the proportion of patients receiving different RDIs by patient characteristics

Outof a total of 503 breast cancer patients, 293 (52% received neochemotherapy) who were treated with TEC regimen and had both case and follow-up records available were included in this study. The characteristics of those patients are shown in Table 1. Most of the patients were under 69 years of age (98%), had AJCC stage II–III disease (69%), received mastectomy (95%), used G-CSF (83%), did not use radiotherapy (22%), and did not have comorbidities (77%). Our comorbidity data showed that 40 patients had coexisting diabetes, 19 had hypertension, and 9 had both. No other coexisting diseases were found in this cohort, and the comorbidity did not affect the RDI received. Fifty-eight percent of the study population was HR(+). Patients that used hormone therapy accounted for 47% of the total population. A total of 266 (91%) patients had a dose reduction, and 80 (27%) patients had chemotherapy delays. Dose reductions were defined as chemotherapy dose reduction ≥ 5% from the NCCN standard. Chemotherapy delays were defined as chemotherapy dose delays ≥ 7 days.

**Table 1:** Proportion of stage I–III breast cancer patients, regardless of subtype, who received a reduced relative dose intensity, stratified by demographic and clinical characteristics, at the First Affiliated Hospital, Chongqing Medical University, 2011.

RDI	ALL		≥ 90%		p	80–90%		p	< 80%		p
**N**	293	%	102	34.8 (%)		110	37.5 (%)		81	27.7 (%)	
**Age**					0.18			0.36			0.12
≤ 49	171	58.4	64	62.75		57	51.82		50	61.7	
50–59	87	29.7	31	30.39		38	34.55		18	22.2	
60–69	30	10.2	7	6.86		13	11.82		10	12.3	
> 69	5	1.7	0	0.0		2	1.82		3	3.7	
**Menopausal status**					0.59			0.06			0.16
Pre-menopause	154	52.6	56	36.4		50	32.5		48	31.2	
Post-menopause	139	47.4	46	33.1		60	43.2		33	23.7	
**AJCC stage**					0.16			0.11			0.03
I	75	25.6	28	37.3		22	29.3		25	33.3	
II	130	44.4	48	36.9		57	43.8		25	19.2	
III	73	24.9	18	24.7		30	41.1		25	34.2	
Data missing	15	5.1	8	53.3		1	6.7		6	40.0	
**T**					0.76			0.003			< 0.001
T1 ≤ 2 cm	110	37.5	41	37.3		31	28.2		38	34.5	
T2 2–5 cm	129	44.0	43	33.3		54	41.9		32	24.8	
T3 > 5 cm	7	2.4	2	28.6		5	71.4		0	0.0	
T4	33	11.3	9	27.3		19	57.6		5	15.2	
Data missing	14	4.8	7	50.0		1	7.1		6	42.9	
**Nodal status**					0.36			0.37			0.002
N0	173	59.0	62	35.8		68	39.3		43	24.9	
N1	66	22.5	25	37.9		28	42.4		13	19.7	
N2	34	11.6	8	23.5		9	26.5		17	50.0	
N3	17	5.8	4	23.5		5	29.4		8	47.1	
Data missing	3	1.0	3	100.0		0	0.0		0	0.0	
**HR status**					0.67			0.07			0.13
HR(+)	169	57.7	59	34.9		57	33.7		53	31.4	
HR(−)	105	35.8	34	32.4		47	44.8		24	22.9	
Data missing	19	6.5	9	47.4		6	31.6		4	21.1	
**HER2/neu status**					0.11			< 0.001			0.009
Positive (HER2(3+)/FISH+)	11	3.8	1	9.1		6	54.5		4	36.4	
Positive (HER2(2+))	146	49.8	46	31.5		69	47.3		31	21.2	
Negative	117	39.9	45	38.5		29	24.8		43	36.8	
Data missing	19	6.5	10	52.6		6	31.6		3	15.8	
**Operation method**					0.40			0.60			0.78
Breast-conserving surgery	16	5.5	4	25.0		7	43.8		5	31.3	
Mastectomy	277	94.5	98	35.4		103	37.2		76	27.4	
**Chemotherapy type**					0.67			0.89			0.55
Adjuvant	140	47.8	47	33.6		52	37.1		41	29.3	
Neoadjuvant	153	52.2	55	35.9		58	37.9		40	26.1	
**Use of G-CSF**					0.00			0.65			< 0.001
Yes	244	83.3	95	38.9		93	38.1		56	23.0	
No	49	16.7	7	14.3		17	34.7		25	51.0	
**Use of hormone therapy**					0.06			0.19			0.02
Yes	139	47.4	61	43.9		47	33.8		31	22.3	
No	135	46.1	32	23.7		56	41.5		47	34.8	
Unknown	19	6.5	9	47.4		7	36.8		3	15.8	
**Radiotherapy**					0.15			0.86			0.11
Yes	65	22.2	18	27.7		25	38.5		23	35.4	
No	228	77.8	85	37.3		85	37.3		58	25.4	
**Comorbidity**					0.10			0.67			0.19
Yes	68	23.2	18	26.5		27	39.7		23	33.8	
No	225	76.8	84	37.3		83	36.9		58	25.8	
**Dose reduction**					< 0.001			0.08			0.01
Yes	266	90.8	83	31.2		104	39.1		79	29.7	
No	27	9.2	19	70.4		6	22.2		2	7.4	
**Chemotherapy delays**					< 0.001			0.03			< 0.001
Yes	80	27.3	6	7.5		22	27.5		52	65.0	
No	213	72.7	96	45.1		88	41.3		29	13.6	

HR: ER, oestrogen receptor and PR, progesterone receptor; HER2, human epidermal-growth factor receptor 2; AJCC, American Joint Committee on Cancer; G-CSF, granulocyte-growth factors/cytokines.

A total of 102 (35%) patients received an RDI more than 90%, 110 (38%) patients received an RDI between 80 and 90%, and 81 (28%) patients received an RDI less than 80%. We found that young patients were more likely to receive a high RDI. The highest proportion of patients who received an RDI ≥ 90% was the ≤ 49 years group; of the patients who were more than 60 years old, only 7 out of 35 patients received an RDI ≥ 90%. However, there were no significant differences, and the sample size was so small that we could only speculate that the older the patient was, the lower the RDI she received.

There were 244 (83%) patients who used G-CSF during chemotherapy. Patients who used G-CSF accounted for 93% (n = 95) of patients who received an RDI ≥ 90%, while those who received RDIs between 80–90% that used G-CSF accounted for 85% (n = 93). The proportion decreased to 69% (n = 56) when patients received an RDI < 80%. The use of G-CSF was the only factor associated with a higher likelihood of receiving an RDI ≥ 90% (p = 0.001).

Patients with stage I or III tumours (p = 0.03), with positive lymph node involvement (p = 0.002) and without G-CSF (p < 0.001) were more likely to receive an RDI < 80%. T4 tumours are unlikely to reduce the RDI (p < 0.001).

### The impact of RDI on disease-free survival and overall survival

**Figure 1:**
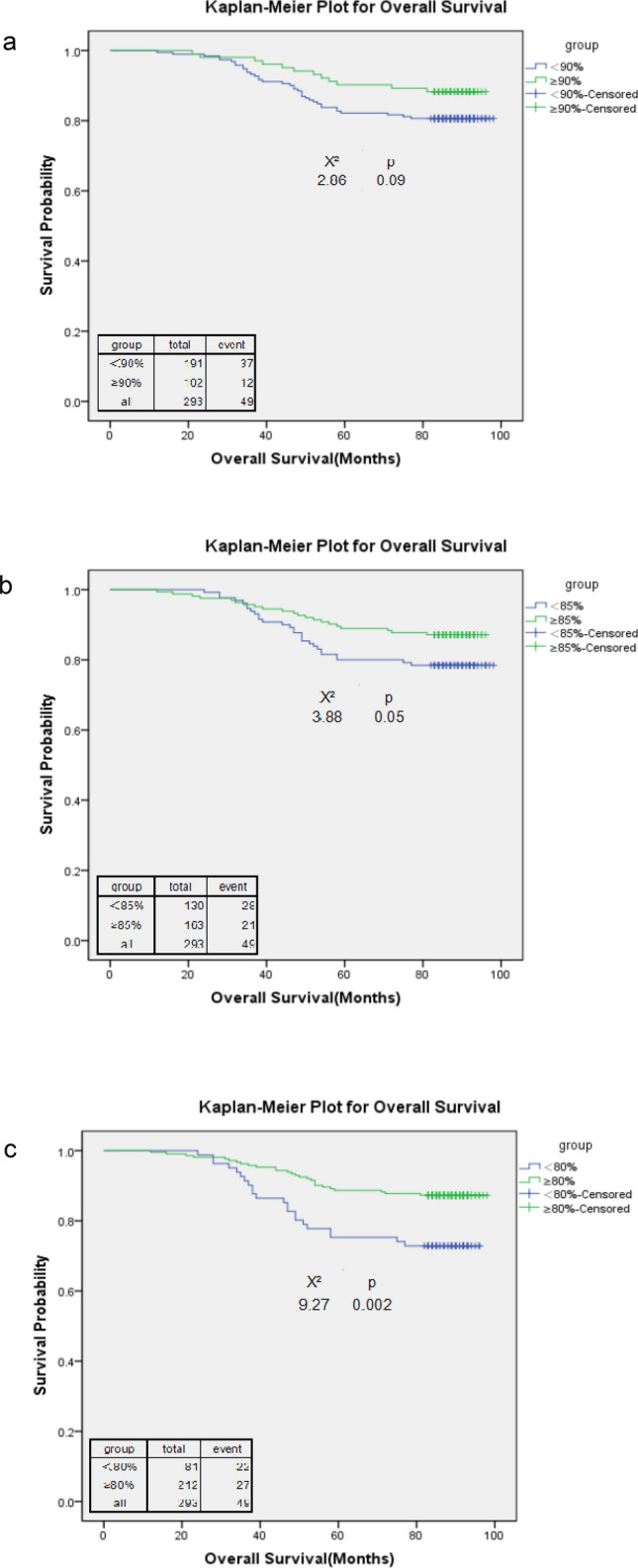
Kaplan–Meier curve for overall survival among stage I–III breast cancer patients, regardless of subtype, with a relative dose intensity (RDI) cut-off point of 90 (**a**), 85 (**b**), and 80 (**c**).

**Figure 2:**
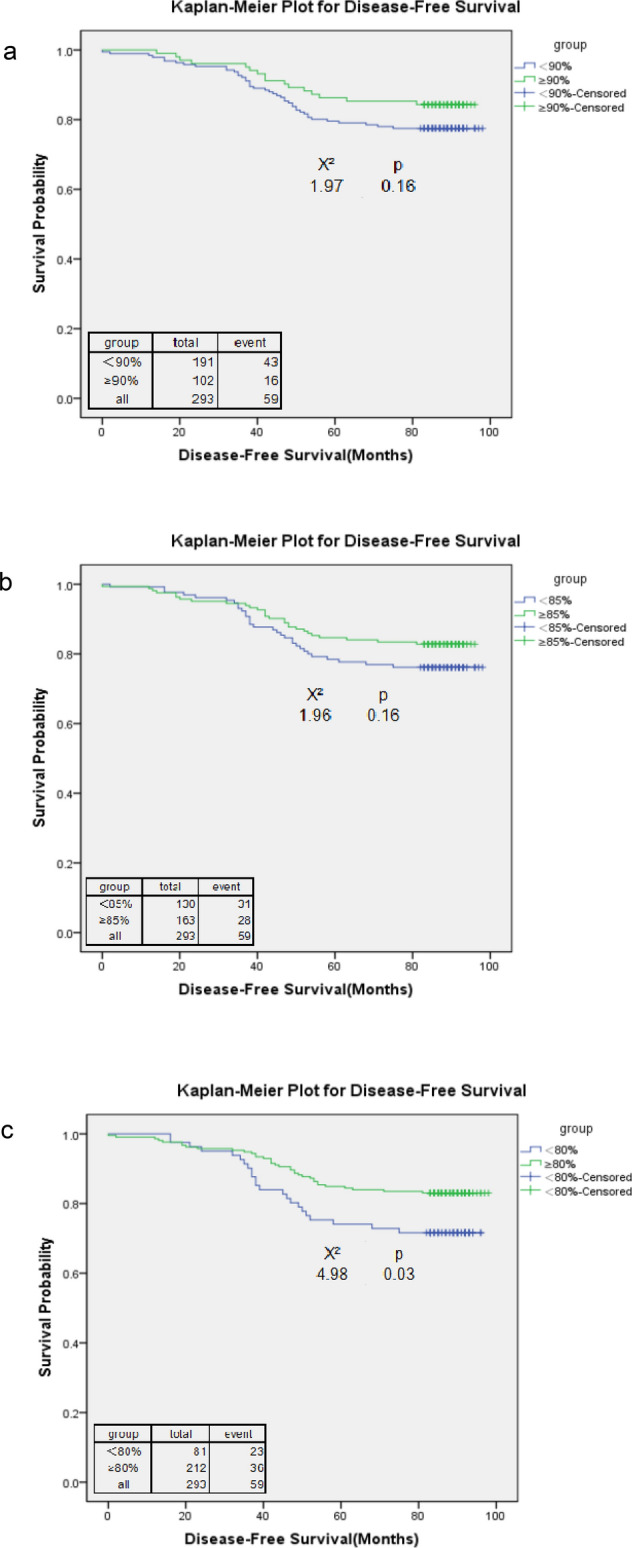
Kaplan–Meier curve for disease-free survival among stage I–III breast cancer patients, regardless of subtype, with a relative dose intensity (RDI) cut-off point of 90 (**a**), 85 (**b**), and 80 (**c**).

Regardless of subtype, 65% of 293 patients (n = 191) had an RDI < 90%, and the number decreased to 28% (n = 81) of patients who received an RDI < 80% (Table 2). The overall survival rate of 293 patients was 83% (median follow-up, 7.33 years), regardless of the RDI level. The overall survival rate was 88% when the RDI ≥ 90%, and the number decreased to 77% when the RDI < 80%. The overall survival rates declined with the RDI levels. The RDI cut-off point of 85% showed a significant overall survival difference (HR = 2.04; 95% CI 1.13–3.70; p = 0.02). However, 80% was the cut-off point at which a high RDI was associated with a significantly better overall survival (HR = 2.61; 95% CI 1.44–4.73; p = 0.002) and disease-free survival (HR = 1.97; 95% CI 1.14–3.42; p = 0.02). The RDI cut-off point of 80% also showed both the highest HR estimate for disease-free survival and overall survival. For both disease-free survival and overall survival, the HR estimates increased with the RDI (Table 2). The Kaplan–Meier survival curves confirmed the survival differences resulting from different RDI cut-off points (Figs. 1 and 2).

**Table 2:** Adjusted hazard ratios (HRs) for time to disease-free survival and overall survival for different cut-off points of the relative dose intensity (RDI) among stage I–III breast cancer patients, regardless of subtype.

RDI	% Reduced RDI (n = 293)	Overall survival rate (%)	DFS	Disease-free survival		Overall survival	
				Adjusted HR	p	Adjusted HR	p
All		83.3	79.9				
RDI < 90% vs. ≥ 90%	65.2 vs. 34.8	80.6 vs. 88.2	77.5 vs. 84.3	1.50 (0.83, 2.72)	0.18	1.74 (0.88, 3.44)	0.11
RDI < 85% vs. ≥ 85%	44.4 vs. 55.6	79.2 vs. 86.5	76.2 vs. 82.8	1.57 (0.93, 2.65)	0.09	2.04 (1.13, 3.70)	0.02
RDI < 80% vs. ≥ 80%	27.7 vs. 72.4	76.5 vs. 85.9	71.6 vs. 83.0	1.97 (1.14, 3.42)	0.02	2.61 (1.44, 4.73)	0.002

Covariates that were adjusted included age at diagnosis, tumour size, lymph node involvement, comorbidity, and use of granulocyte-growth factors/cytokines.

### Impact of RDI on survival for breast cancer patients by HR status

For HR(+) breast cancers and HR(−) breast cancers, covariates that were adjusted included age at diagnosis, tumour size, lymph node involvement, comorbidity, and use of granulocyte-growth factors/cytokines. For HR(+) breast cancers, the use of hormone therapy was also adjusted.

**Figure 3:**
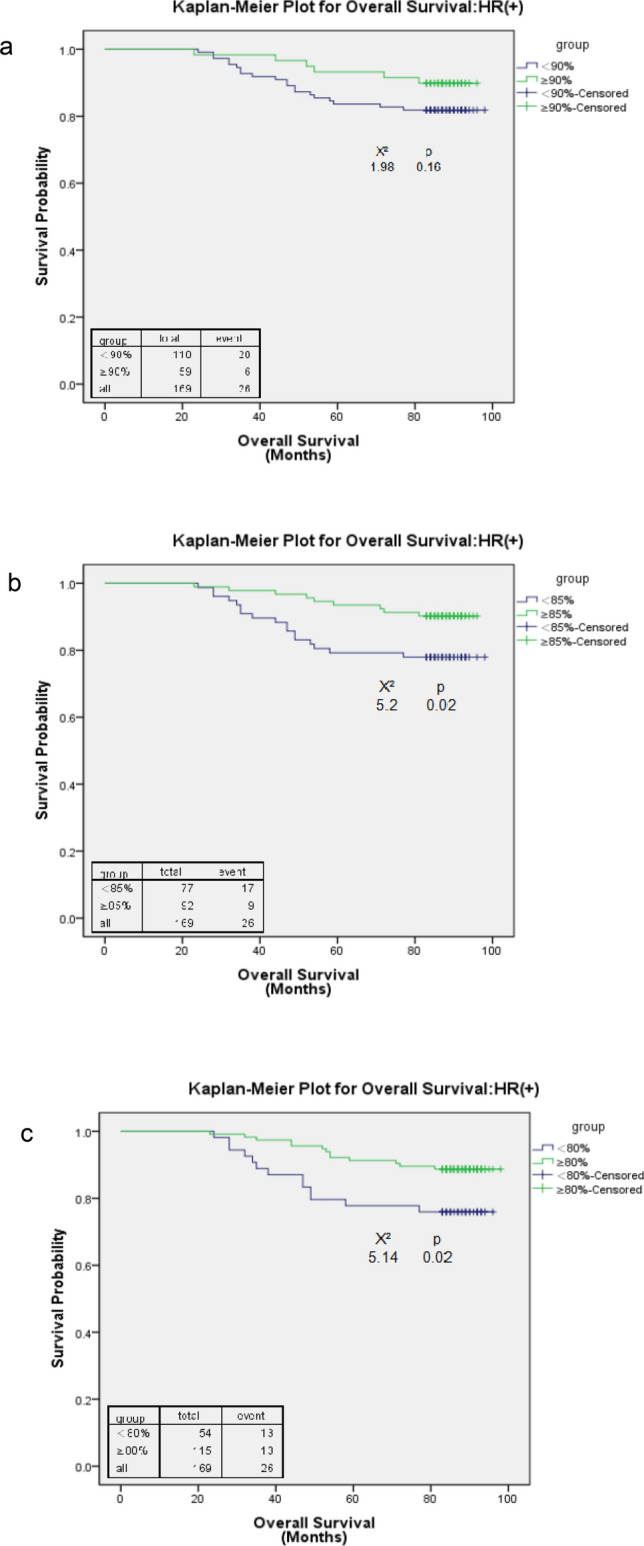
Kaplan–Meier curve for overall survival among stage I–III oestrogen receptor- or progesterone receptor-positive (HR+) breast cancer patients with a relative dose intensity (RDI) cut-off point of 90 (**a**), 85 (**b**), and 80 (**c**).

**Figure 4:**
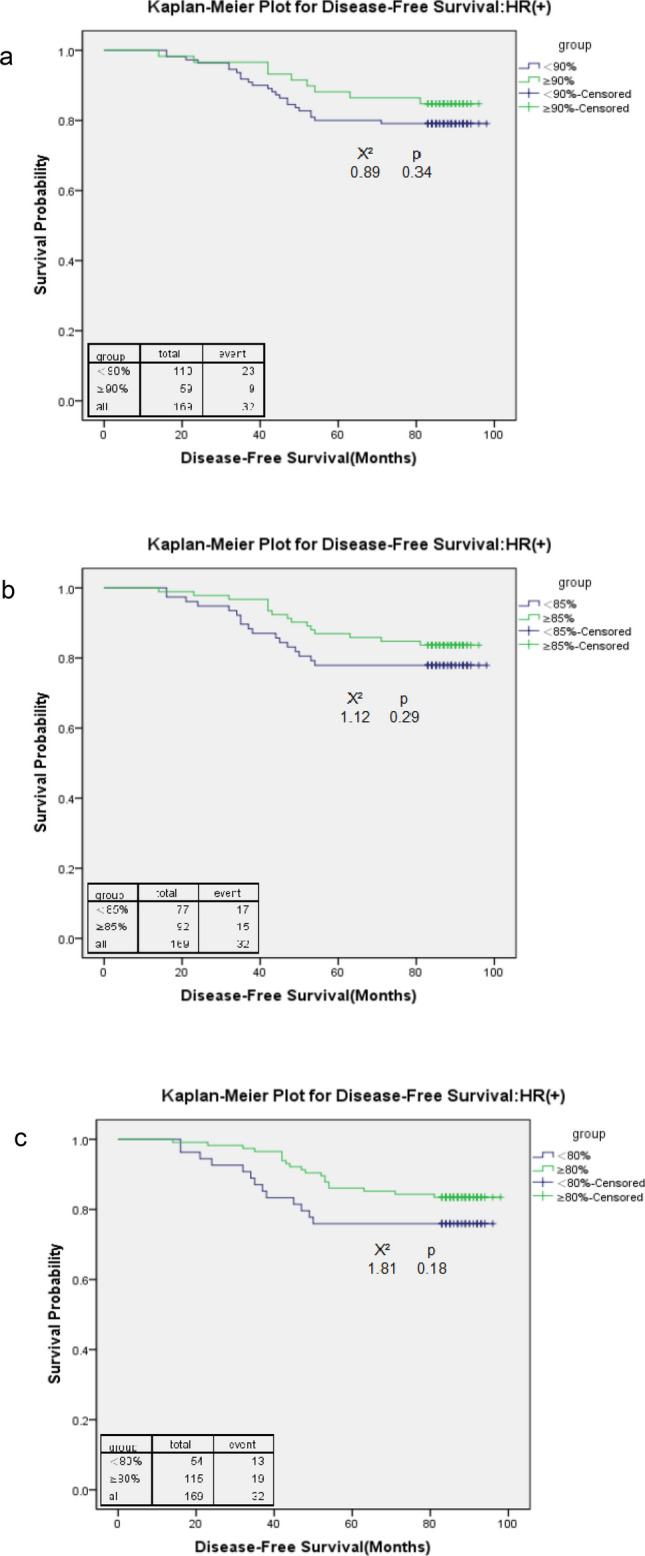
Kaplan–Meier curve for disease-free survival among stage I–III oestrogen receptor- or progesterone receptor-positive (HR+) breast cancer patients with a relative dose intensity (RDI) cut-off point of 90 (**a**), 85 (**b**), and 80 (**c**).

A total of 110 HR(+) patients (65%) who received an RDI < 90%, and the number of patients who received an RDI < 85% decreased to 77 (46%). Only 54 (32%) HR(+) patients received RDI < 80% (Table 3). Among HR(+) patients, both an RDI of 85% (HR = 3.00, 95% CI 1.24–7.26; p = 0.02;) and 80% (HR = 2.92, 95% CI 1.25–6.82; p = 0.01) were the cut-off points at which the higher RDI was associated with a significantly better overall survival. An RDI of 80% (HR = 2.33; 95% CI 1.07–5.08; p = 0.03) was the only cut-off point for DFS. At all cut-off points, a higher RDI was associated with a better survival estimate (HR > 1). The Kaplan–Meier survival curves confirmed the survival differences resulting from different RDI cut-off points (Figs. 3 and 4). Both the RDI cut-off points of 80% and 85% showed apparent survival benefits from receiving a high RDI for OS. However, no DFS benefits were observed when patients received a high RDI at any cut-off point.

**Table 3:** Adjusted hazard ratios (HRs) for time to disease-free survival and overall survival at different cut-off points of the relative dose intensity (RDI) among stage I–III oestrogen receptor- or progesterone receptor-positive (HR+) breast cancer patients and oestrogen receptor- and progesterone receptor-negative (HR−) breast cancer patients.

RDI	N	% reduced RDI	Disease-free survival		Overall survival	
			Adjusted HR	p	Adjusted HR	p
**HR(+) (N = 169)**						
RDI < 90% vs. ≥ 90%	110 vs. 59	65.1	1.62 (0.72, 3.65)	0.24	2.26 (0.84, 6.04)	0.10
RDI < 85% vs. ≥ 85%	77 vs. 92	45.6	1.81 (0.86, 3.81)	0.12	3.00 (1.24, 7.26)	0.02
RDI < 80% vs. ≥ 80%	54 vs. 115	32.0	2.33 (1.07, 5.08)	0.03	2.92 (1.25, 6.82)	0.01
**HR(−) (N = 105)**						
RDI < 90% vs. ≥ 90%	71 vs. 34	67.6	2.16 (0.72, 6.43)	0.17	2.22 (0.63, 7.78)	0.22
RDI < 85% vs. ≥ 85%	46 vs. 59	43.8	1.59 (0.68, 3.72)	0.29	1.45 (0.55, 3.81)	0.45
RDI < 80% vs. ≥ 80%	24 vs. 80	22.9	2.07 (0.85, 5.07)	0.11	2.86 (1.05, 7.80)	0.04

For HR(+)and HR(−) breast cancer patients, covariates that were adjusted included age at diagnosis, tumour size, lymph node involvement, comorbidity, and use of granulocyte-growth factors/cytokines. For HR(+) breast cancers, the use of hormone therapy was also adjusted.

A total of 71 HR(−) patients (67.6%) received an RDI < 90%, and the number of patients who received an RDI < 85% decreased to 46 (44%).Only 24 (23%) HR(−) patients received an RDI < 80% (Table 3).

**Figure 5:**
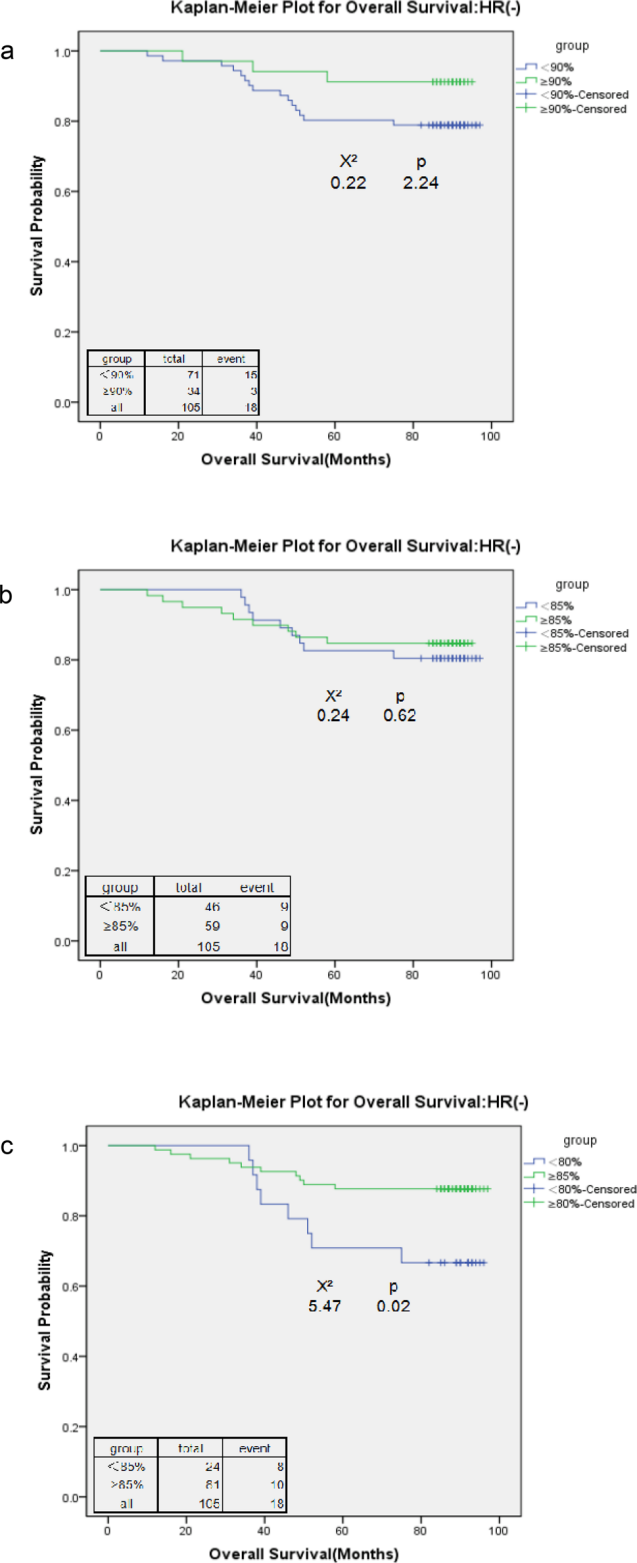
Kaplan–Meier curve for overall survival among stage I–III oestrogen receptor- and progesterone receptor-negative (HR−) breast cancer patients with a relative dose intensity (RDI) cut-off point of 90 (**a**), 85 (**b**), and 80 (**c**).

**Figure 6:**
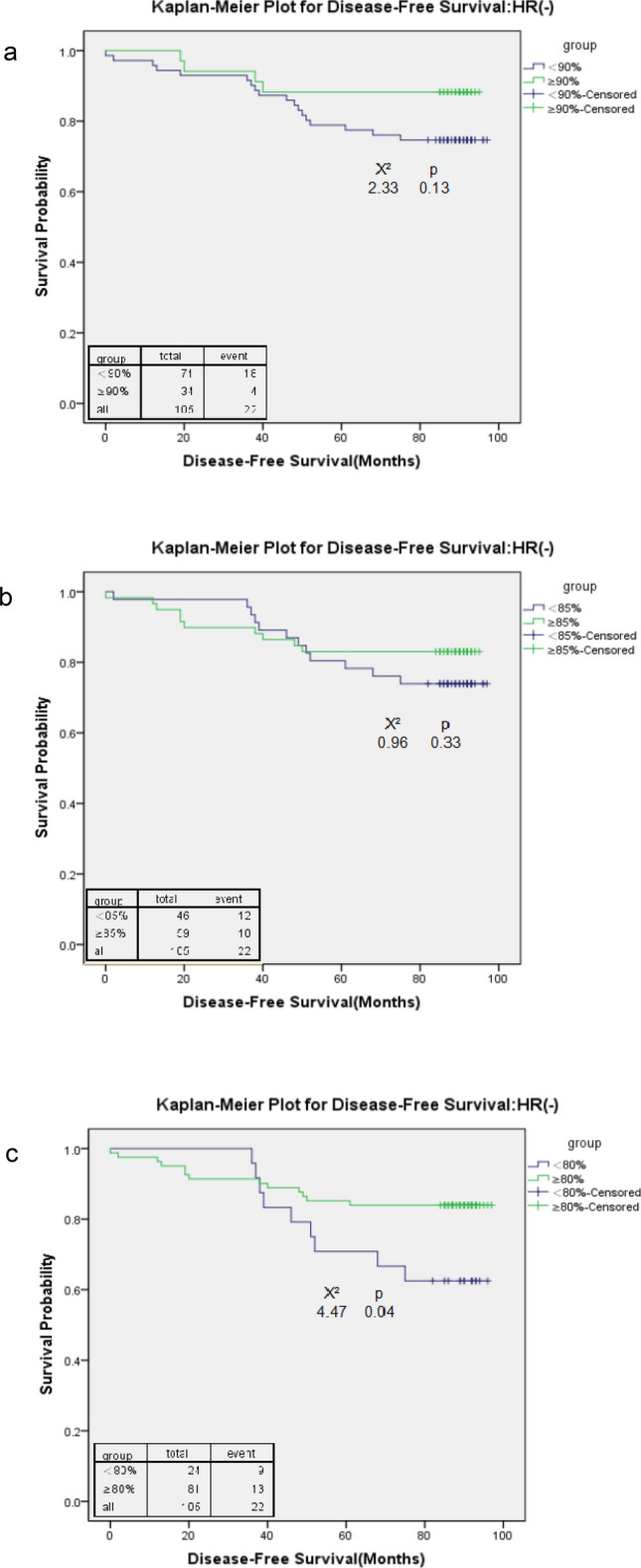
Kaplan–Meier curve for disease-free survival among stage I–III oestrogen receptor- and progesterone receptor-negative (HR−) breast cancer patients with a relative dose intensity (RDI) cut-off point of 90 (**a**), 85 (**b**), and 80 (**c**).

Among HR(−) patients, 80% was the only cut-off point at which the higher RDI was significantly associated with better overall survival (HR = 2.86; p = 0.04) (Table 3). However, no DFS benefits were observed from receiving a high RDI at any cut-off point. Similar to HR(+) patients, each cut-off point was associated with HR > 1 for both DFS and OS of HR(−) patients. The Kaplan–Meier survival curves confirmed the survival differences (Figs. 5 and 6). An RDI of 80% was associated with the most contrasting survival differences for both OS and DFS outcomes, whereas at other cut-off points, no survival benefits were observed from receiving a high RDI.

### The impact of RDI on adverse events

Myelosuppression was the major toxicity. There were documented varying degrees of neutropenia in 249 patients (85%), most of which (n = 158) were grade 3–4. Among the 249 patients with neutropenia, 96 (39%) received an RDI ≥ 90%, 55 (22%) received an RDI between 85 and 90%, 40 (16%) received an RDI between 80 and 85%, and 58 (23%) received an RDI ≤ 80% (Table 4). The incidence significantly decreased as the RDI decreased (p = 0.03). Twenty-two patients experienced febrile neutropenia out of the 249 neutropenia patients. The occurrence of anaemia was much less than that of neutropenia, and most of the anaemia experienced was grade 1–2. Thrombocytopenia was rare, and only 5 out of 293 patients experienced thrombocytopenia.

**Table 4:** The side effects of maintaining a high RDI compared with a low RDI for stage I–III breast cancer patients.

Toxicity type	N	Grade^a^	≥ 90% (n = 102)	85%-90% (n = 61)	80%-85% (n = 49)	< 80% (n = 81)	p
**Haematological toxicity**							
Anaemia	55	1–2	15 (14.7)	11 (18.0)	10 (20.4)	14 (17.3)	0.59
		3–4	0	3 (4.9)	0	2 (2.5)	
Neutropenia	249	1–2	36 (35.3)	20 (32.8)	15 (30.6)	20 (24.7)	0.03
		3–4	60 (58.8)	35 (57.4)	25 (51.0)	38 (46.9)	
Thrombocytopenia	5	1–2	1 (1.0)	0	1 (2.0)	2 (2.5)	0.71
		3–4	1 (1.0)	0	0	0	
**Cardiac toxicity**	14	2	4 (3.9)	1 (1.6)	3 (6.1)	6 (7.4)	0.41
**Diarrhoea**	23	1–3	11 (10.8)	5 (8.2)	4 (8.2)	3 (3.7)	0.37
**Nausea**	160	1–3	66 (64.7)	34 (55.7)	25 (51.0)	35 (43.2)	0.01
**Vomiting**	129	1–3	56 (54.9)	27 (44.3)	20 (40.8)	26 (32.1)	0.02
**Hepatic disorders**	33	1–3	11 (10.8)	7 (11.5)	10 (20.4)	5 (6.2)	0.1
**FN**	22		5 (4.9)	4 (6.6)	7 (14.3)	6 (7.4)	0.23

FN, febrile neutropenia.

^a^The classification of side effects is based on the Common Terminology Criteria for Adverse Events, version 4.02.

As shown in this table, a large number of patients experienced nausea (n = 160) and vomiting (n = 129). A higher RDI of chemotherapy related to a higher probability of nausea (p = 0.01) and vomiting (p = 0.02). Other side effects were relatively few. There were 14 patients who experienced grade 2 cardiac toxicities, 23 patients had diarrhoea episodes and 33 patients had hepatic disorders, and these adverse events were not correlated with the level of RDI. The reason may be related to the small sample size of these side effects that limited the ability to achieve statistical significance.

## Discussion

Our study investigated the TEC regimen-specific cut-off points of RDI. We found the optimized chemotherapy RDI cut-off points of the TEC regimen were associated with improved disease-free and overall survival for stage I–III breast cancer, which suggested that a higher RDI was associated with a better survival for both disease-free and overall survival. To optimize overall survival benefits for all patients regardless of subtype, RDI should be maintained ≥ 85% and for disease-free survival, RDI should be maintained ≥ 80%. And 80% was the cut-off point at which a high RDI was associated with both a significantly better overall survival and disease-free survival; specifically, the RDI should be ≥ 85% in HR(+) patients and ≥ 80% in HR(−) patients.

Our results were similar but not exactly the same as what was found decades ago. Colleoni et al. analysed 10-year follow-up data from four RCTs starting during 1978–1993^21^ and aimed to assess the effect of adjuvant CMF in premenopausal women with lymph node-positive breast cancer. They found that a CMF dose level < 85% was associated with a significantly worse 10-year disease-free and overall survival only for patients with ER(−) tumours but not for patients with ER(+) tumours^21^. In addition, two other old studies recommend 85% as the optimal RDI cut-off point with the previous generation regimens of CMF and CAF for early-stage breast cancer^8,22^. However, those previous generation regimens have long been substituted with the new generation regimens that contain anthracycline combined with docetaxel. The clinical trial NSABP B15 should be a start for this substitution, which proved that regimen doxorubicin and cyclophosphamide (AC) leads to the same disease-free survival and overall survival as regimen CMF^23^. Later, CAGLB 9344 provided evidence that AC-T improves both DFS and OS by 6% compared with regimen AC, and a receptor-negative cohort could obtain more benefits from AC-T than from AC^5^. In 2006, BCIRG001 started and finally showed that TAC improves both DFS and OS by 7% compared with regimen FAC after a 7-year follow-up^24^, and the Intergroup Study 0148 report justified the adjuvant use of taxanes after an anthracycline-containing regimen^16^; since then, the TAC regimen has become widely accepted in China. Doctors have been using taxanes combined with anthracycline as a classical regimen for the past decade. Therefore, our research used the new generation regimen containing anthracycline combined with docetaxel and was based on the Chinese population, which could be a reason for the different results from the old studies.

BCIRG005 suggested that the survival benefits of the two regimens were similar, but the incidence of grade 3/4 adverse events in the blood system was lower in the AC-T regimen than in the TAC regimen^6^. However, the AC-T regimen had not been accepted well in Western China due to its longer duration than that of TAC, especially for neoadjuvant chemotherapy at that time.

Over the past decades, people have taken maintaining a high RDI as a goal of chemotherapy administration^10,25^. However, a higher RDI can lead to higher toxicity rates, which cause a reduction in compliance. In addition, some side effects are life-threatening. To avoid toxicity in some patients, physicians must compromise by using a lower RDI. The ultimate goal of breast cancer management is to achieve personalized treatment by maximizing benefits while minimizing unnecessary risks. Our results suggested that when patients received a regimen containing anthracycline and taxanes, it was possible to modify their chemotherapy RDI.

Our study has several limitations. First, this study was a retrospective study, and we were not able to obtain complete information for patient characteristics, as some data were missing. For example, the FISH examination of HER2 and trastuzumab treatment were not covered by insurance in 2011, which led to HER2(2+) without a FISH exam in half of the population; therefore, we cannot define classical subtypes for every patient and cannot evaluate the effect of RDI among patients with HER2-positive or HER2-negative tumours. Second, the small sample size could have limited the ability to achieve statistical significance of survival differences for certain RDI cut-off points. Third, we were not capable of investigating the relationship between cause-specific death and RDI because the reason for death is not transparent. Last but not least, the adverse reaction data used in this study were based on patients’ memory, and a previous electronic medical records system may result in inaccurate data. These detailed analyses could be more informative for personalized treatment.

In conclusion, we observed improved survival for an increased chemotherapy RDI for both HR(+) and HR(−) breast cancer patients among breast cancer patients diagnosed in 2011 in our hospital. To maintain survival benefits, an RDI should be maintained ≥ 85% for HR(+) patients and ≥ 80% for HR(−) patients. We also found that a higher RDI of chemotherapy relates to a higher probability of neutropenia, nausea and vomiting. Evidence from our single study may not be sufficient to determine an actual RDI limit for each subtype or to identify how much RDI can be compromised when severe toxicity occurs; however, our study raised a doubt that the standard dosage of chemotherapy is applicable for all patients in China. Studies with larger, more heterogeneous patient populations are needed to validate our results and the chemotherapy RDI cut-off points by different breast cancer subtypes.

## Data Availability

All data included in this study are available upon request by contacting the corresponding author, Qiao Cheng.
